# Antibacterial Biocomposite Based on Chitosan/Pluronic/Agarose Noncovalent Hydrogel: Controlled Drug Delivery by Alginate/Tetracycline Beads System

**DOI:** 10.3390/jfb15100286

**Published:** 2024-09-28

**Authors:** Hossein Abdollahi, Saber Amiri, Farzaneh Amiri, Somayeh Moradi, Payam Zarrintaj

**Affiliations:** 1 Department of Polymer Engineering, Faculty of Engineering, Urmia University, Urmia 57561-51818, Iran; somayemooradii75@gmail.com; 2 Department of Food Science and Technology, Faculty of Agriculture, Urmia University, Urmia 57561-51818, Iran; sa.amiri@urmia.ac.ir; 3 Department of Pharmaceutics, Faculty of Pharmacy, Tabriz University of Medical Science, Tabriz 51666-53431, Iran; amirif1234@gmail.com; 4 Department of Biomedical and Pharmaceutical Sciences, University of Montana, Missoula, MT 59812, USA; payam.zarrintaj@gmail.com

**Keywords:** biocompatible polymer, composite hydrogel, antibacterial, wound dressing, sustained drug release

## Abstract

Designing a wound dressing with controlled uptake, antibacterial, and proper biocompatibility is crucial for the appropriate wound healing process. In this study, alginate/tetracycline (Alg/TC) beads were produced and embedded into chitosan/pluronic/agarose semi-interpenetrating polymer network hydrogel, which serves as a potential biocompatible dressing for treating skin wounds. The effect of pluronic content on the porosity, swelling, mechanical characteristics, and degradation of the hydrogel was investigated. Furthermore, the impact of Alg beads on TC release was subsequently examined. In the absence of Alg beads, faster release was observed. However, after incorporating beads into the hydrogels, the release was sustained. Particularly, the hydrogel containing Alg beads exhibited a nearly linear release, reaching 74% after 2 days in acidic media. The antimicrobial activity and biocompatibility of the hydrogel were also evaluated to assess the capability of the TC-loaded hydrogels for wound dressing applications. The hydrogel demonstrated efficient antibacterial features against Gram-positive and Gram-negative bacteria. Additionally, the sample behavior was evaluated against exposure to yeast. Furthermore, based on biocompatibility studies using HFF2 cells, the TC-loaded hydrogel exhibited remarkable biocompatibility. Overall, this novel composite hydrogel shows remarkable biocompatibility and antibacterial activities which can be used as a great potential wound dressing to prevent wound infections due to its effective inhibition of bacterial growth.

## 1. Introduction

Creating an optimal environment is crucial for the complex and dynamic process of wound healing. Wound dressings play a vital role in wound care as they protect the wound from infection and aid in its recovery. Evolving from simple wound coverings, modern wound dressings are designed to facilitate wound healing by establishing a suitable milieu that inhibits bacterial infection and promotes cell adhesion and proliferation [1,2].

From a general perspective, wound dressings can be classified into two main groups. The first group comprises conventional wound dressings that are applied to the wound bed to provide protection. These dressings make the wound anhydrous and cannot stimulate the healing process. In contrast, the second group consists of modern dressings, which come in a variety of materials tailored to specific wound types. Modern dressings not only create an optimal environment for the healing process but also possess enhanced antibacterial properties and maintain moisture in the wound area. In addition, to facilitate effective wound recovery, an ideal dressing should possess several key attributes. It should have the capacity to absorb excess wound secretions, retain the necessary moisture for wound healing, allow gas exchange, be flexible enough to conform to the body’s shape, and exhibit suitable mechanical strength. A more favorable approach involves utilizing a system capable of maintaining an effective concentration of an antimicrobial drug, such as tetracycline (TC), at the wound site for a specified duration [3,4,5].

Biocompatible hydrogels have gained considerable attention as a preferred material for wound dressings. These hydrogels possess an interconnected and spongy structure that is beneficial for tissue regeneration [6]. As previously mentioned, the ability to maintain moisture in the wound area is crucial for facilitating the healing process, and hydrogels excel in this regard due to their excellent water-absorbing properties. Additionally, hydrogels with controlled release capabilities have the potential to enhance the healing rate by incorporating various growth factors, antibacterial agents, and even cells [7,8].

One of the commonly employed materials for preparing hydrogels is chitosan (Chit). Chit-based hydrogels are considered ideal for wound dressing due to their biodegradability, biocompatibility, and broad-spectrum antimicrobial activities. These hydrogels promote cell adhesion, proliferation, and differentiation owing to their hydrophilic nature and similarity to the extracellular matrix [2,9]. When utilized as a wound dressing, Chit-based hydrogels rapidly absorb wound exudate, creating an optimal environment for cellular activity, preventing bacterial proliferation, and fostering cell growth. However, one limitation of using Chit alone is its poor mechanical properties, which can affect its integrity. The weak mechanical properties have been identified as major drawbacks in the application of neat Chit dressings [10].

In recent decades, the technique of combining multiple types of polymers has emerged as a means to modify the mechanical properties of polymers, offering advantages over single-component systems. Agarose (Aga), a biocompatible natural polysaccharide with a thermos-gelling feature and satisfactory mechanical performance, has been extensively used as a matrix material in various studies for the preparation of wound dressings. The porous structure of Aga gel facilitates nutrient delivery, making it favorable for wound healing applications. Additionally, the hardness of Aga gel can be easily adjusted by altering the polymer concentration [11,12]. Studies have indicated that Aga gel exhibits limited cell adhesiveness and cell proliferation activity. Consequently, there has been an increasing trend in blending Aga with other polymers, such as Chit, to overcome these drawbacks. The combination of these polymers results in the formation of hydrogen bonds between the functional groups of both polymers, allowing Chit chains to be incorporated into the Aga network. This leads to an improvement in the elastic modulus and overall mechanical properties. Research on the blending of Aga and Chit has demonstrated promising outcomes [13].

In recent years, there has been significant interest in utilizing pluronics (Plrs) as nonionic surfactants with amphiphilic structures for drug delivery purposes. Plrs have gained attention due to their ability to facilitate sustained drug release upon gelation near body temperature. However, the use of Plr hydrogels faces challenges, including their dissociation and loss of integrity in aqueous environments, leading to rapid drug permeation and the inability to achieve desired controlled delivery over an extended duration. To address this challenge, scientists have recently made attempts to develop novel composite systems [14,15,16].

Based on the aforementioned considerations, in this study, we propose the incorporation of antibiotic-loaded beads into composite hydrogels as a solution to achieve desirable mechanical performance and controlled release. Therefore, the objective of our current research is to design and prepare reinforced Chit/Plr/Aga (CPA) composite hydrogels, capitalizing on the advantageous properties of Chit in proper biocompatibility and pH sensitivity, Aga in enhancing the mechanical properties of hydrogels, and Plr in controlling drug release. Additionally, alginate beads were utilized to investigate their effect on drug release within the composite system. To the best of our knowledge, these specific systems have not been previously examined. The samples were analyzed for structural and morphological characteristics, swelling ratio, in vitro degradation, tetracycline (TC) release behavior, and mechanical strength. Furthermore, the antibacterial properties of TC-loaded composite hydrogels were assessed. As a result, the prepared pH-responsive hydrogels offer a promising strategy for promoting skin wound healing.

## 2. Materials and Methods

### 2.1. Materials

Agarose (Aga, MW~120 kDa, low gel temperature between 26–30 °C), sodium alginate (Alg, MW~120–200 kDa), chitosan (Chit, 75–85% degree of deacetylation, MW~190–310 kDa), pluronic F-127 (Plr, MW approximately 12.5 kDa, melting point around 55.4 °C, boiling point > 149 °C), and tetracycline hydrochloride (TC, molar mass 444.4 g/mol, melting point 172 °C) were all purchased from Sigma-Aldrich (St. Louis, MO, USA). In addition, calcium dichloride (CaCl_2_) and acetic acid (AcOH) were obtained from Merck (Darmstadt, Germany).

### 2.2. Synthesis

#### 2.2.1. Synthesis of Alg/TC Beads

**Scheme 1:**
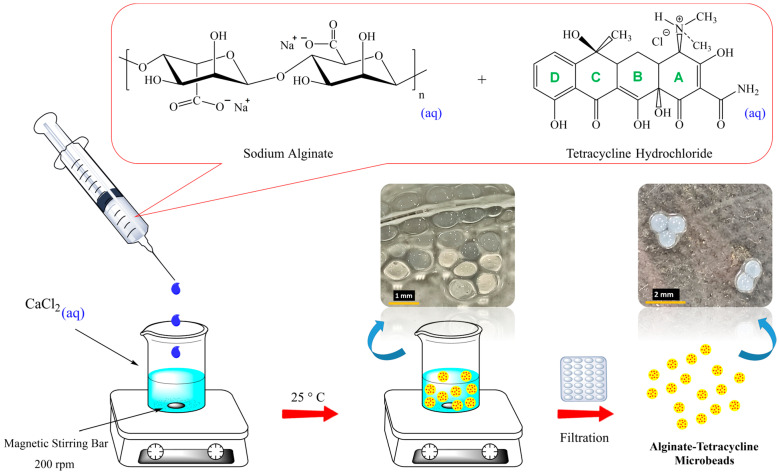
Simplified schematic of alginate/tetracycline beads preparation process.

To synthesize Alg/TC beads, 0.1 gr of Alg and 0.05 gr of TC were separately dissolved in 10 cc of DI water. The resultant solutions were combined and agitated at ambient temperature for 2 h, yielding a transparent solution. Then, using a syringe pump with a flow rate of 0.5 μL min−1, this solution was added to the 10% CaCl_2_ solution, which served as a gelling agent. When droplets fell into the CaCl_2_ solution, a chemical crosslinking reaction was initiated through the interaction with calcium ions in the medium, and beads were formed. Magnetic stirring was employed to facilitate the reduction in the size of the resulting beads. For enhanced clarity, a schematic of the preparation process for the Alg/TC beads is presented in Scheme 1.

#### 2.2.2. Characterization of Alg/Tc Beads

The encapsulation efficiency of tetracycline was assessed by suspending beads in an appropriate volume of the phosphate-buffered saline (PBS) with continuous mixing. After 24 h, a part of encapsulated tetracycline was released. Then, by broking the beads using ultrasonic for 3 min the complete release of TC was achieved, and the concentration of encapsulated drug was determined by UV/Vis spectrophotometry at a wavelength of 266 nm. The measurements were conducted in triplicate. The ratio of the determined mass of TC which was released from the beads and the mass of TC at first introduced into the beads demonstrates the encapsulation efficiency and it was about (79.85 ± 1.50%).

The bead size was evaluated by examining the diameter of 50 distinct beads under a standard inverted-light microscope. The size of the beads was measured using ImageJ software (1.53 t). Beads with a diameter of 950 ± 30 µm (mean ± s.d., *n* = 50) were successfully produced.

#### 2.2.3. Synthesis of Chit/Plr/Aga (CPA)-Based Hydrogels

First, 0.1 g of Chit was dissolved in 10 cc of distilled water by adding 1% acetic acid and the mixture was stirred for 1 h at room temperature. Simultaneously, a specific amount of Plr was added to 10 cc of distilled water and mixed for 30 min. Then, the obtained solutions were combined and mixed for another 30 min. Afterward, the pre-prepared Alg/TC beads were incorporated into the samples. Finally, 0.1 g of Aga was added to 10 cc of boiling water. After the Aga was dissolved, the solution was added to the samples to form the final hydrogels.

To investigate the influence of Plr content on the mechanical, swelling, and biological properties, three samples were prepared with different amounts of Plr (0, 0.05, and 0.1 g). The samples were labeled as CPAMi-0.0, CPAMi-0.05, and CPAMi-0.1 when Alg/TC beads were included. The samples without Alg/TC beads were labeled as CPA-0.0, CPA-0.05, and CPA-0.1, respectively.

### 2.3. Characterization

#### 2.3.1. Structural Characterization

Fourier transform infrared (FT-IR) analysis was applied to analyze the hydrogel. FT-IR spectroscopy (ThermoNicolet Nexus 670, Waltham, MA, USA), running at a resolution of 4 cm^−1^ in the range of 4000 to 400 cm^−1^.

#### 2.3.2. Microstructure Investigations

The morphologies of the hydrogels were assessed by utilizing scanning electron microscopy (Vega SEM, Tescan Company, Brno, Czech Republic). Hydrogels were freeze-dried and gold sputtered. The pore sizes of hydrogels were estimated from SEM micrographs by Digimizer, software (Version 5.4.9).

In addition, the sample’s porosity was evaluated using the liquid displacement technique. In this regard, freeze-dried samples were submerged in a determined volume of ethanol (*V*_1_). The total volume of ethanol and hydrogel (*V*_2_) was determined. Then, hydrogels were removed and the remaining volume of ethanol (*V*_3_) was determined. Subsequently, porosity was calculated utilizing Equation (1) [17]:

(1) $$\varepsilon \%=\frac{V_{1}-V_{3}}{V_{2}-V_{3}}\times 100$$

#### 2.3.3. Swelling Behavior

Determined amounts of samples were submerged in PBS solutions (pH: 5.8, 6.8, 7.4, and T: 37 °C). At defined times, hydrogels were taken out of the submerging solution and weighed after cleaning their surface water until reaching equilibrium weight. The swelling ratio (SR) was calculated utilizing Equation (2) [18]:

(2) $$\mathrm{SR}\%=\frac{W_{s}-W_{d}}{W_{d}}\times 100$$

where *W_s_* and *W_d_*, are the weight of the swollen hydrogel at a specific time interval and dry gel weight, respectively. The experiment was performed in triplicate.

#### 2.3.4. In Vitro Study of the Hydrogel Degradation

The samples were submerged in PBS (pH 7.4) and kept at 37 °C for a specific time. After 1, 3, 7, 14, and 21 days, the samples were removed and washed up with DI water. Then, the samples’ weight was calculated to evaluate the degradation rate (DR) using Equation (3) [19]:

(3) $$\mathrm{DR}\%=\frac{W_{0}-W_{1}}{W_{0}}\times 100$$

where *W*_0_ and *W*_1_ are the weight of the initial gel, and the weight of gel after being immersed in PBS, respectively. All experiments were performed in triplicate.

#### 2.3.5. Mechanical Property

To assess the hydrogels’ mechanical performance, tubular shapes with 10 mm diameter and 5 mm thickness were made. Uniaxial compression was employed on the hydrogels utilizing the Texture Analyser TAXT Plus (Stable Micro System, Surrey, UK) with a 5 kN load cell and 1 mm·min^−1^ strain rate to determine the stress–strain curve.

#### 2.3.6. In Vitro Drug Release Assay

The loading of TC in the CPA hydrogels was confirmed in FTIR analysis. To study the in vitro release profile of TC, the prepared samples were immersed in a chamber containing PBS (100 rpm, pH: 5.8, 6.8, 7.4, and 37 °C) as a release medium to imitate the physiological condition. Then, 1 mL of medium was taken out at the specified time and changed with the equivalent amount of fresh medium to maintain the volume constant. The concentration of the drug was evaluated by UV–vis spectrophotometry (WPA, Cambridge, UK) using a wavelength of 357 nm to measure the drug release pattern. All experiments were repeated three times. To assess TC release, the correlation between TC concentration and UV absorption was analyzed by the standard curve.

#### 2.3.7. Evaluation of the Antibacterial Activity of Hydrogel

##### Microbial Strains

Gram-positive bacteria (*Staphylococcus aureus* ATCC 29213, *Staphylococcus saprophyticus* ATCC 15305 and *Enterococcus faecalis* ATCC 29212), Gram-negative bacteria (including *Escherichia coli* ATCC 25922, *Klebsiella pneumoniae* ATCC 7881, *Salmonella typhimurium* ATCC 14028 and *Pseudomonas aeruginosa* ATCC 27853) and yeast (*Candida albicans* ATCC 10231) were utilized to determine antimicrobial activity.

##### Determining the Minimum Inhibitory Concentration and Minimum Bactericidal Concentration

The minimum inhibitory concentration (MIC) of hydrogels was defined through the micro broth dilution method which conformed to the Clinical and Laboratory Standards Institute (CLSI, 2021) guidelines VET04. Bacterial strains were cultured and incubated during night at 37 °C in 5 mL brain heart infusion (BHI) broth (Merck, Germany) and diluted to 5 × 10^5^ colony forming unit (CFU)/mL. Also, *C. albicans* were cultured at 30 °C for 48 h in 5 mL Sabouraud-Dextrose Broth (SDB) (Merck, Germany) and diluted to 5 × 10^5^ colony forming unit (CFU)/mL. The stock concentrations of hydrogels were diluted in distilled water to obtain concentrations in the range of 3.125, 6.25, 12.5, 25, 50, 100 and 200 μg/mL.

200 μL of Mueller–Hinton broth (Merck, Germany) medium containing 100 μL of hydrogels (3.125, 6.25, 12.5, 25, 50, 100 and 200 μg/mL) was added in polypropylene 96-well microplate. Then, 100 μL of the prepared bacterial suspension of 0.5 McFarland standard (5 × 10^5^ CFU/mL) was added to the wells and then kept for 18 h at 37 °C in the incubator. After incubation, the absorbance was evaluated by a spectrophotometer at 550 nm.

The wells containing only Mueller–Hinton broth and Mueller–Hinton broth with microorganisms were utilized as negative and positive controls, respectively. The MIC was described as the lowest possible concentration that prevented visible bacterial growth observed within the microplate (no turbidity).

Minimum bactericidal concentration (MBC) was measured by re-inoculation of broth dilutions applied to determine MIC on agar media. Briefly, 30 μL of broth dilutions of MIC was cultured onto Mueller–Hinton agar (Merck, Germany) medium and incubated overnight at 37 °C. The lowest broth dilution of hydrogels that inhibits the bacteria growth on the agar plate is known as MBC. The lack of bacterial growth in the Mueller–Hinton agar was indicative of MBC.

#### 2.3.8. In Vitro Evaluation of Hydrogel Cytotoxicity

Methyl thiazolyl tetrazolium (MTT) assay was performed to assess the biocompatibility of fabricated hydrogels. In this context, HFF2 cells were incubated with different concentrations of developed hydrogels and their possible cytotoxicity was determined based on previously published research [20]. Hydrogel was incubated with cultured media and then cells were treated with different concentrations of incubated cell culture media. The typical procedure of MTT assay is as follows: in 96-well plates, after the confluence of the HFF2 cells reached the required density (8000 cells/well), they were treated with extracted hydrogels media (at varied concentrations) ranging from 15.625 to 10,000 μg/mL. After 48 h of incubation, the culture medium was aspirated and 20 μL of MTT reagent (5 mg/mL) was poured into each well. After 4 h incubation, the solution was aspirated and 100 μL DMSO was poured into every well and the absorbance of each of them was read at 570 nm utilizing an ELISA reader. Ultimately, the cell viability percentage was determined by comparison of the optical density (OD) with the control group utilizing Equation (4) (results were stated as mean ± SD):

(4) $$\mathrm{Viability}\;\mathrm{Rate}\;\%=\frac{{\mathrm{OD}}_{\mathrm{Hydrogel}}}{{\mathrm{OD}}_{\mathrm{Control}}}\times 100$$

#### 2.3.9. Evaluation of Hydrogel Hemocompatibility

1 mL of 2% pre-washed blood was incubated with 660 μL of PBS containing 340 mg of hydrogels at 37 °C for 4 h. After incubation, the mixtures were centrifuged, and the OD of the supernatant was recorded. Triton X-100 and PBS were used as the positive and negative control, respectively.

#### 2.3.10. Statistical Analysis

The results are analyzed statistically to investigate significant differences among different samples. GraphPad Prism 9 software, version 9.3.0 was used to perform statistical analysis by one-way ANOVA and *t*-test. The data were expressed as a mean ± standard deviation and the *p*-value of less than 0.05 is considered statistically significant.

## 3. Results and Discussion

### 3.1. FT-IR Characterization

**Figure 1:**
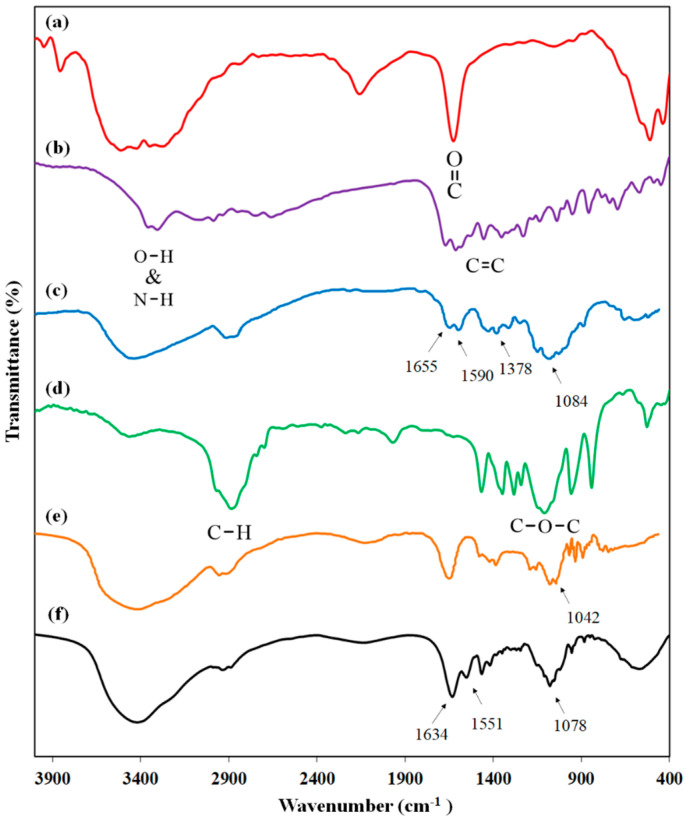
FT-IR patterns of (**a**) sodium alginate, (**b**) tetracycline, (**c**) chitosan, (**d**) pluronic, (**e**) agarose, and (**f**) CPAMi hydrogel.

**Scheme 2:**
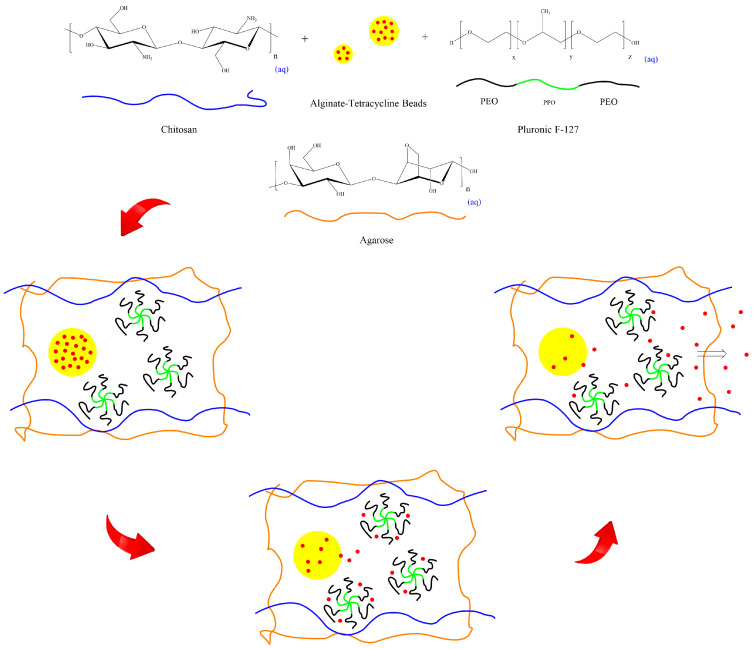
Schematic illustration of the drug release mechanism for CPAMi hydrogel systems.

Characterization of the CPA hydrogel containing Alg-TC drug carrier beads (CPAMi) systems was monitored first in FT-IR analysis. The FT-IR spectra of the Alg, TC, Chit, Plr, Aga, and final hydrogel in the region 400–4000 cm^−1^ were presented in Figure 1. Also, the molecular structure is depicted in Scheme 2.

Figure 1a illustrates the FT-IR spectrum of Alg. The spectrum demonstrates a wide band in the range of 3100–3600 cm^−1^ attributed to O–H stretching vibration. The specific absorption peaks at 1626 cm^−1^ presented in the spectrum of Alg correspond to COO^−^ stretching vibration. In addition, the peak observed at 2158 cm^−1^ could be attributed to the CO_2_ groups [21].

In the case of TC, Figure 1b, the bands at around 3400 cm^−1^ were attributed to hydroxyl and amine groups. The peaks at 1670 and 1531 cm^−1^ are related to the carbonyl and amino groups of the amide in ring A, respectively. The 1613 and 1583 cm^−1^ peaks were designated to the carbonyl groups in A and C rings, respectively (the position of the rings is shown in Scheme 1). The absorption peak at 1454 cm^−1^ was for the aromatic -C=C- skeletal vibration [22].

Figure 1c is the spectrum of Chit powder. Here, it is obvious that the stretching of intra- and intermolecular O–H overlapped with stretching NH_2_ vibrations at 3440 cm^−1^. The peaks at 2874 cm^−1^ relate to the asymmetric C–H vibrations. The vibrational mode of amide I, II, and III was observed at 1655, 1590, and 1378 cm^−1^, respectively. The absorption peak of C-O-C groups was revealed at 1084 cm^−1^, while the small peak at around 900 cm^−1^ is attributed to the wagging of the saccharide structure of Chit [23].

In the spectrum of Plr, Figure 1d, sharp bands can be seen at around 1350 cm^−1^ allocated to the wagging vibrations of the CH_2_ groups; the CH_2_ twisting vibrations give rise to two bands at 1242 and 1282 cm^−1^, whereas the existence of the methyl groups is proved by the peak at 1467 cm^−1^. In addition, the bands at 2885 cm^−1^ are related to the C–H stretching vibration. The C–O–C stretching vibrations give rise to the main peak at about 1100–1000 cm^−1^; lastly, the peaks at 958 and 842 cm^−1^ are designated as CH_2_ rocking bands [24].

The FTIR spectrum of Aga is given in Figure 1e. This spectrum shows O–H stretching and intermolecular hydrogen bonds along with bending at 3429 cm^−1^ and 1640 cm^−1^, respectively. The asymmetric and symmetric modes of CH_2_ were detected at 2950 and 2878 cm^−1^, respectively. The bands at 921 cm^−1^ fit the 3,6-anydro-β-galactose structure and the bands at 1042 cm^−1^ could be related to the C–O–C groups [25].

The FTIR spectrum corresponding to the CPAMi-0.05 sample, Figure 1f, showed the characteristic bands of each species, which indicates the preparation of the CPAMi hydrogel systems. For instance, this spectrum shows the characteristic absorption bands of TC (e.g., carbonyl and aromatic -C=C- skeletal absorption bands), confirming the successful loading of TC in the hydrogel. However, when various materials are blended, the changes in specific peaks of the FTIR spectrum could indicate the chemical interactions. The amide II band observed at 1590 cm^−1^ in Chit (Figure 1c) shifted to 1551 cm^−1^ (Figure 1f) indicating intermolecular hydrogen bonding between the Chit amino groups and Aga hydroxyl groups. Also, the absorption bands at 1084 cm^−1^ (Figure 1c) and 1042 cm^−1^ (Figure 1e) associated with O–C–O stretching joined to become one single band at 1078 cm^−1^ (Figure 1f), suggesting the presence of many hydrogen bonds and microphase separation [26].

### 3.2. Microstructure Assessments

**Figure 2:**
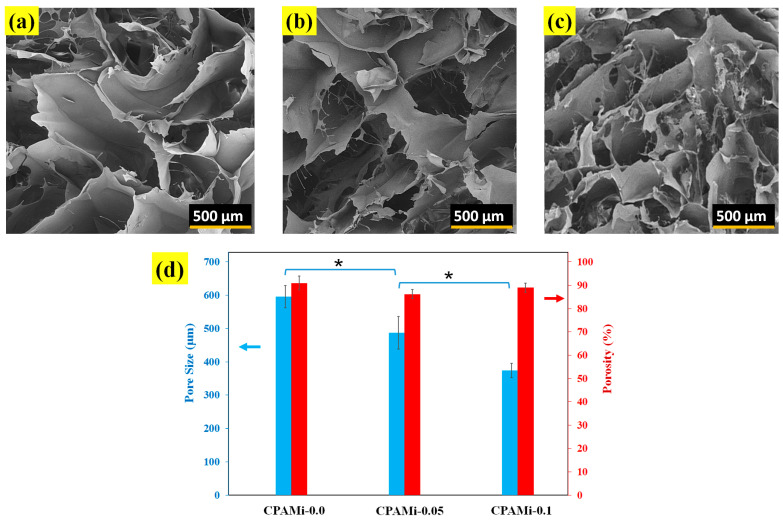
SEM images of (**a**) CPAMi-0.0, (**b**) CPAMi-0.05, and (**c**) CPAMi-0.1 hydrogels and (**d**) attributed pore size and porosity bar charts for the samples. * represent significant difference *p* < 0.05.

Tissue-engineered 3-D microstructures should have a porous nature appropriate for cell ingrowth and for exchange of the nutrients, metabolites, and debris. To observe the morphology of the hydrogels and the impact of the Plr concentrations on their structures, the prepared hydrogels were freeze-dried and their pore size was studied using SEM imaging. The appearance and the microstructure under SEM of the hydrogels are displayed in Figure 2.

As seen in Figure 2a–c, SEM micrographs demonstrate an open-cell morphology with a completely porous and interconnected 3-D structure for hydrogels, which upon the addition of Plr, denser structures were obtained. The pore size of all the hydrogels was measured using Digimizer and the average pore sizes of the samples are given in Figure 2d. The CPAMi-0.0 hydrogel showed larger pore sizes compared to the samples with Plr. The pore sizes of the hydrogels decreased considerably (*p* < 0.05) from 596 to 487 and 374 μm by adding 0.05 and 0.1 g of the Plr to the systems, respectively.

It can be hypothesized that the intermolecular interactions in hydrogel play the main role in the formation of the porous structure [27,28]. Plr is an amphiphilic copolymer with hydrophobic propylene oxide (PPO) and hydrophilic polyethylene oxide (PEO) chains [29]. When Pluronic (Plr) is introduced into an aqueous medium, the hydrophobic PPO chains reduce their interaction with the surrounding water, while the hydrophilic PEO chains form hydrogen bonds with the water molecules. This interaction affects the arrangement of the polymer chains and influences the overall porous structure of the hydrogel network. As the concentration of Pluronic increases, the entanglement between polymer chains becomes more pronounced, leading to a reduction in pore size. These structural changes impact the hydrogel’s ability to control drug release [13,30,31].

The porosity of the hydrogels was measured by the liquid displacement technique and also depicted in Figure 2d. According to the results, the porosities are 91%, 86%, and 89% for the CPAMi-0.0, CPAMi-0.05, and CPAMi-0.1 hydrogels, respectively. As can be seen, the statistical analysis suggested no significant difference among the samples in terms of porosity percentage (*p* > 0.05). One of the effective factors in the porosity of hydrogels is polymer-to-solvent concentration. Therefore, it could be said that because the same polymer-to-solvent concentration was used in the samples, the porosities of different samples were close to each other.

The pore size, or the size of the void spaces in a material, is also important for cell growth. If the pore size is tiny, it can restrict the diffusion of nutrients and oxygen, leading to reduced cell growth. If the pore size is too large, cells may not be able to attach and proliferate effectively. The ideal pore size for cell growth also depends on the type of cells and the conditions they require. In the context of cell growth, porosity is important because it determines the availability of nutrients and oxygen that are necessary for cell proliferation and survival. High porosity allows for better diffusion of these essential substances, while low porosity can lead to reduced cell growth. Moreover, with proper pore size distribution, cells can properly adhere, proliferate and migrate. In the literature, it is stated that pore sizes greater than 300 μm [32] and porosity between 60–90% [33] are favorable for wound healing applications as they are capable of providing sufficient space for cell proliferation, tissue ingrowth, and oxygen and nutrient exchange. Hence, based on the achieved pore size and porosity, it can be claimed that the present hydrogel pore size and porosity are suitable for biological activities.

In the SEM analysis, the images obtained revealed the porous network structure of both the hydrogel and the bead. The SEM images provided visual evidence of the interconnected pores within the hydrogel, indicating its porosity and potential for fluid absorption and retention. Similarly, the bead displayed a porous structure, as observed in the SEM images.

**Figure 3:**
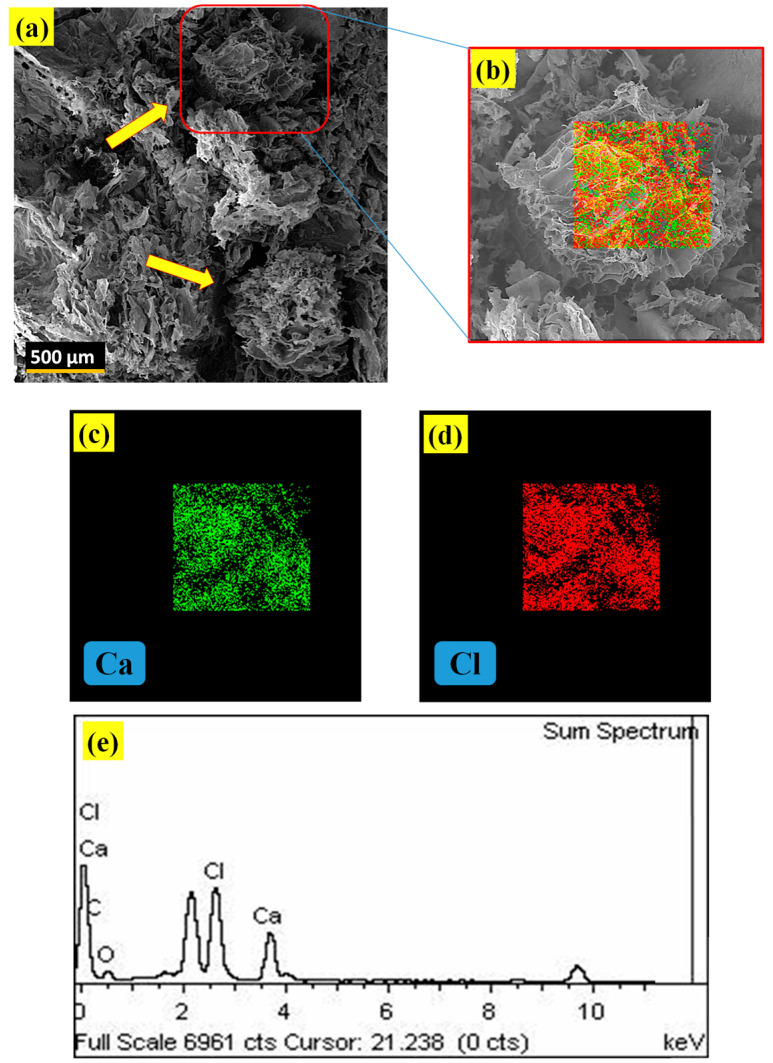
Microscopic analysis of sample CPAMi-0.1 (**a**) SEM surface micrograph, (**b**) SEM micrograph of alginate/tetracycline bead, (**c**) Ca mapping, (**d**) Cl mapping, and (**e**) EDS elemental pattern.

Furthermore, EDX analysis was performed to investigate the elemental composition of the bead. The results from the elemental analysis (Figure 3) confirmed the presence of calcium (Ca) and chlorine (Cl) in the bead, which were used in the crosslinking process. The presence of these elements indicated the successful incorporation of the crosslinking agents within the bead structure.

The SEM and EDX analyses collectively provided valuable insights into the morphology and elemental composition of both the hydrogel and the bead, supporting their characterization and potential application in wound healing and related biomedical fields.

### 3.3. Swelling Behavior of the CPAMi Hydrogels

**Figure 4:**
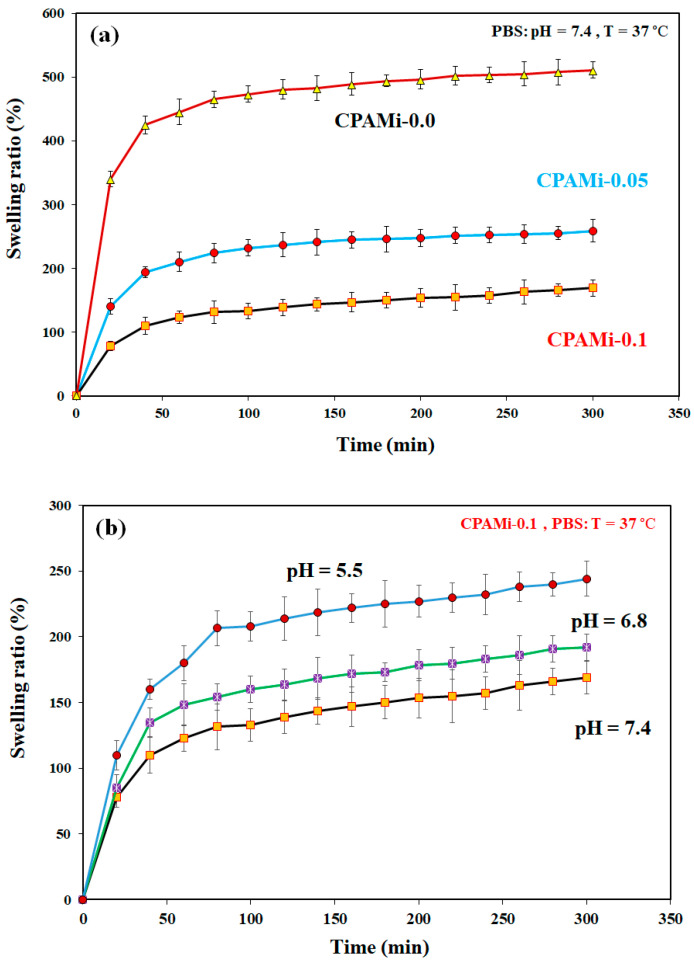
(**a**) The swelling ratio of the CPAMi samples in PBS (pH: 7.4 and T: 37 °C). (**b**) Swelling response of CPAMi-0.1 hydrogel in different pH solutions (T: 37 °C).

The swelling ratio is an essential feature of hydrogels because it not only indicates the possible absorption of fluids (especially in wound dressings) but also many vital properties of hydrogels, such as mechanical features and drug release which are dependent on their swelling behavior [31]. Figure 4a illustrates the swelling behavior of the CPAMi samples versus time at PBS solution (pH: 7.4, T: 37 °C).

As observed in Figure 4, the swelling results reveal that all CPAMi samples soaked up PBS fast at the initial time and then had an equilibrium state. The swelling behavior of hydrogels is a time-dependent process that can be affected by different factors such as osmotic pressure and elasticity. The swelling rate of a hydrogel is related to the balance between the uptake and elastic recovery rate. At the beginning of swelling, the rate of PBS uptake is high, and the hydrogel swells quickly. However, as the gel network expands and the concentration of solutes inside the gel decreases, the uptake rate slows down, resulting in a slower swelling rate. Additionally, as the hydrogel swells, the increased mechanical stress caused by the swelling can activate elastic recovery, reducing the degree of swelling. The interplay between swelling, osmotic pressure, and elasticity is a complex and dynamic process that can vary with time, resulting in different swelling behaviors for different hydrogel systems and conditions [34].

A comparative study in the trend of changes in the swelling ratio of the hydrogels shows that the incorporation of Plr in the CPAMi systems leads to reduce the swelling ratio and the Plr-free sample had a significantly higher swelling ratio than others (*p* < 0.05) [35].

In this research, Aga chains are anticipated to form a gel-like structure and positively charged Chit chains entangled into the Aga matrix forming semi-interpenetration networks (semi-IPN). The Plr molecules are placed in the free space formed by the Chit/Aga semi-IPN systems. As a result of this phenomenon, the free volume between polymer chains in the network is occupied, which could reduce the absorption capacity of the hydrogel. On the other hand, the introduction of the Plr content in the hydrogel networks causes the systems to be rigid. The formation of rigid structure led also to the reduction in absorption capacity. Regarding these two phenomena, the observed swelling behavior of the samples could be explained. In the CPAMi hydrogels, the incorporation of Plr causes decreases in the absorption capacity, thus CPAMi-0.1 with higher Plr content reveals the lower swelling ratio [36].

The polymers comprising pH-responsive hydrogels are high-molecular polymers that undergo a volume transition when the pH value of the external environment changes. Thanks to these properties, appropriate polymer selection can affect the swelling of hydrogels in mediums of different pH values and thus influence the drug release profile in the desired mediums. In order to investigate the effect of pH on swelling, this test was performed in the media with different pHs. The swelling results of developed hydrogel in different buffers against time are shown in Figure 4b. The swelling ratio of the CPAMi-0.1 hydrogel at pH = 5.8 increased to 250% after 300 min, which was significantly higher than the swelling ratio at neutral pH (*p* < 0.05). Chitosan has pH-sensitive properties due to the protonation–deprotonation of the free amino groups. Acidic media promotes the protonation of amino groups (–NH_2_) of the chitosan to the ammonium ions (–NH_3_^+^). Electrostatic repulsive forces and a decrease in osmotic pressure at lower pH due to the presence of more ionic groups caused an increase in swelling. As the pH increases, the hydrogel swelling ratio also decreases which was attributed to the deprotonation of the ammonium ions.

### 3.4. Degradation Evaluation of the CPAMi Systems

**Figure 5:**
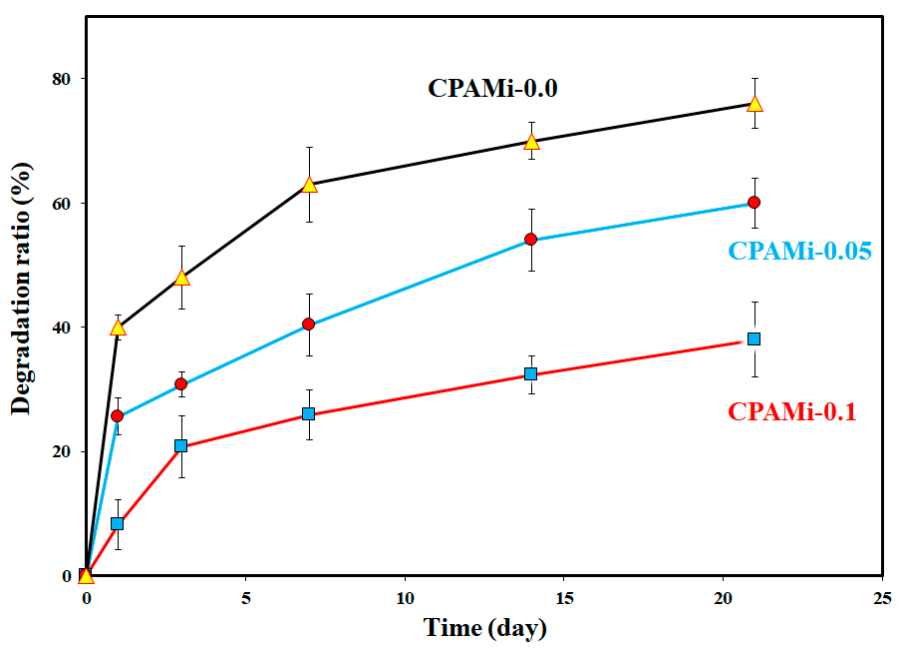
Degradation rate of CPAMi hydrogel systems with various compositions.

The hydrogel degradation rate is a crucial parameter in tissue engineering since chronic inflammation happens because of the long-term existence of scaffolds within the body. Thus, the degradation rate of them should be coordinated with tissue regeneration [35]. The degradation ratio of the CPAMi hydrogel over time is given in Figure 5.

The stability factors of the network are secondary chemical interactions (e.g., hydrogen bonding and hydrophobic force) and physical chain entanglements. Overcoming these intermolecular forces in the entangled network, the chains are released, and the network starts to collapse. When the network is placed in the simulated physiological environment (temperature 37 °C), thermal energy leads to chain mobility and reversible softening of the Chit and Aga in the hydrogel. This phenomenon causes the penetration of water in the network to intensify and the network becomes soft and very swollen [37,38,39]. Then, after some time, the secondary chemical interactions between the polysaccharide chains which depend on the temperature, dissociate [40]. As a result, the chains in the hydrogel relax, which could lead to their release of them from the network. In a similar case, Gómez-Mascaraque et al. [41] have shown that Chit chains are released from the network first. In the last stage, the chain entanglements are slowly untied and the polymer network collapses. As can be seen in Figure 5, the degradation does not alter linearly with time and is more intense in the first days. This trend could be due to the fact that the main forces maintaining the network are secondary interactions. At the temperature of 37 °C, the mobility of the chains and dissociation of the secondary interactions cause a considerable part of the hydrogel structure to separate from the network in the first days. The residual network remains stable due to physical chain entanglements, which also open over time due to defects created in the network and slippage of chains. Maybe, for this reason, the degradation process is observed with less intensity in the following days.

Furthermore, as per Figure 5, all hydrogels show similar behavior, but they do not have the same degradation rate, which is related to the Plr content. As mentioned earlier, Plr molecules occupy the free space of the Chit/Aga network and also cause the network to rigid, which primes to limit further water penetration. Considering that the first stage of the degradation mechanism depends on water penetration, it can be concluded that more Plr content leads to more resistance of hydrogel against degradation. Therefore, compared with the sample CPAMi-0.0, CPAMi-0.1 has a significantly lower degradation rate (*p* < 0.05) due to its low water absorption.

### 3.5. Mechanical Properties of the Hydrogels

**Figure 6:**
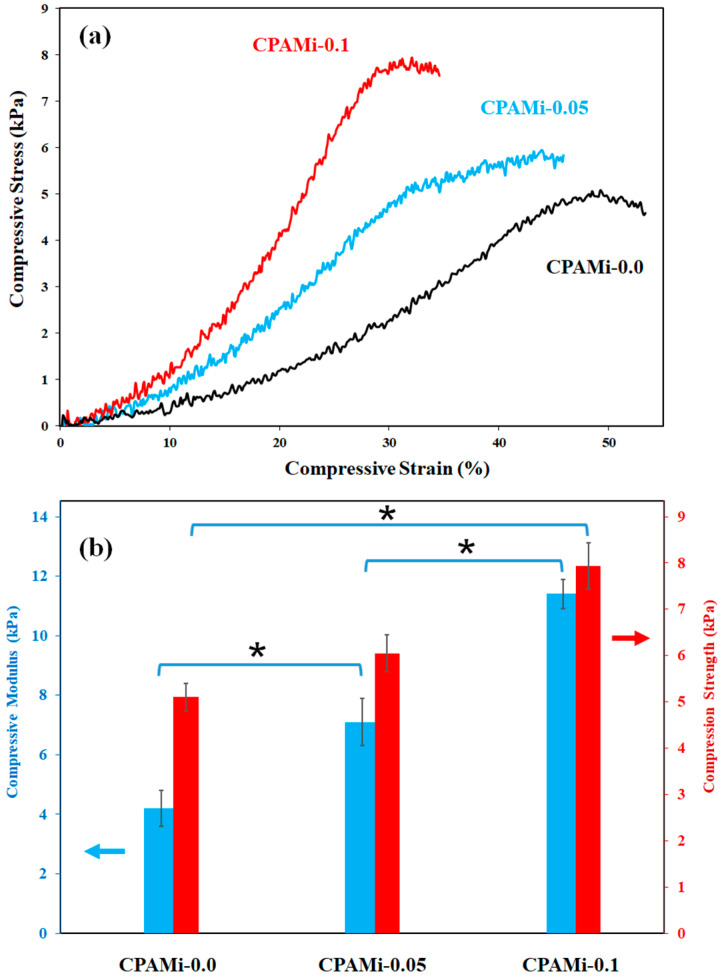
Compression experiment: (**a**) Compressive stress–strain curves, and (**b**) Compressive properties of the CPAMi hydrogels. * represent significant difference *p* < 0.05.

The mechanical features of the scaffolds have an essential role in tissue regeneration, the creation of cell integration, and the ability to resist stress. Here, the mechanical features of the CPAMi samples were determined by the compression test, and the results are demonstrated in Figure 6.

#### 3.5.1. Compressive Properties of the Hydrogel

In this work, it was observed that the primary Chit/Plr hydrogel was an unstable hydrogel, which transformed into a stable hydrogel by adding Aga. The intertwining of Aga chains in semi-IPN networks with Chit chains and hydrogen bonding between Chit and Aga was the speculated reason for the significant enhancement in mechanical properties. As seen in Figure 6a, the compressive stress–strain curves of the hydrogels correspond to the behavior of non-linear and viscoelastic solids [42]. During loading, the entangled polymer chains become reoriented by absorbing the load, the hydrogel is squeezed, and the interstitial water molecules are released from the gel structure. For this process, a small load is sufficient for considerable deformation (i.e., there is no substantial stress up to 10% strain) because, under wet conditions, water not only has a plasticization effect but also causes the weakening of the hydrogen bonding between Chit and Aga [31]. However, as loading persists, the reorientation tends to be uniform, and friction because of the interstitial fluid results in the hardening effect of the polymer, which requires an extra effort to raise the strain.

The stress–strain curves of the CPAMi-0.05 and CPAMi-0.1 samples show an initial rise of compressive stress compared with Plr-free hydrogel. This observation is related to the filling of free spaces in the network by Plr molecules which try to arrest the free movement of polymer chains and make hydrogel rigid (see Scheme 2). The above phenomenon could be one of the reasons for the changes in the mechanical features of the samples due to the addition of the Plr to the systems.

Another reason for changes in mechanical properties can be seen as changes in the morphology of the samples. The mechanical performance depends on the porous nature of the hydrogels [31,43]. As shown in the bar charts in Figure 6b, there were significant differences in compressive moduli of the CPAMi-0.0, CPAMi-0.05, and CPAMi-0.1 hydrogels which were 4.2, 7.1, and 11.4 kPa, respectively. In addition, the CPAMi-0.0 hydrogel shows the least compression strength of 5.10 kPa with a significant deformation of 52%. The compression strength was increased up to 7.94 kPa for hydrogel with 0.1 g of Plr which was significantly higher than the compression strength of CPAMi-0.0 hydrogel (*p* < 0.05). The increase in compressive moduli and strengths of the CPAMi-0.05 and CPAMi-0.1 hydrogels in comparison with the Plr-free sample could be ascribed to the decrease in the pore size (see Figure 2). The observations related to the microstructure section confirm the results obtained in this part.

**Figure 7:**
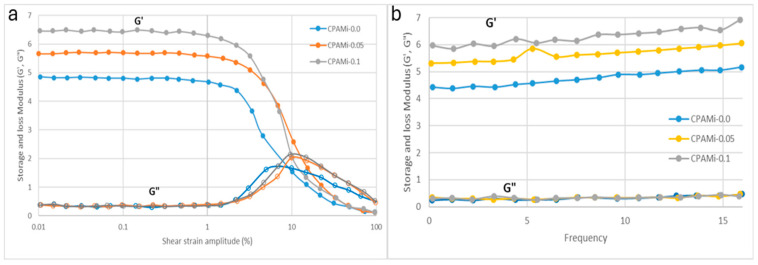
(**a**) amplitude-scan and (**b**) frequency-scan of hydrogel.

To assess the durability of the hydrogels for practical applications, amplitude-scan and frequency-scan rheology were conducted. As shown in the results (Figure 7), the storage modulus (G′) remained consistently higher than the loss modulus (G″) across a wide range of strains and frequencies, indicating the solid-like behavior and mechanical integrity of the hydrogels. Notably, the CPAMi-0.1 hydrogel exhibited the highest storage modulus, demonstrating superior mechanical stability and resilience under mechanical deformation. This suggests that the incorporation of Pluronic enhances the structural rigidity of the hydrogel, making it more durable for practical applications such as wound dressing, where maintaining mechanical strength under stress is crucial.

#### 3.5.2. Shape Recovery Capability of the Hydrogel

**Figure 8:**
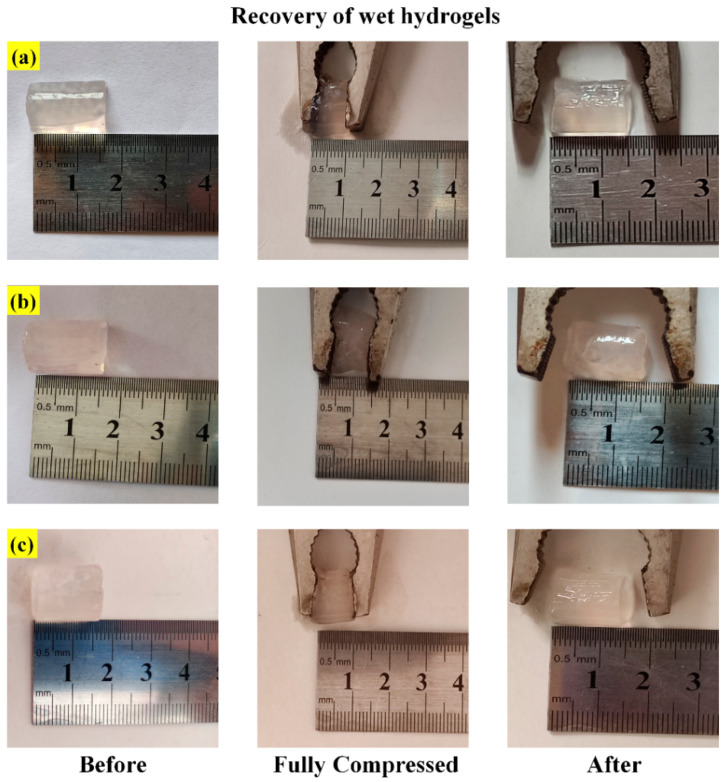
Images exhibit the recovery profiles of (**a**) CPAMi-0.0, (**b**) CPAMi-0.05, and (**c**) CPAMi-0.1 hydrogels. All Samples showed instant recovery after full compression using pliers.

The recovery performance (shape-memory) of hydrogels was also tested. The macroporous sponge-like structure allows the water to flow out/in freely, giving the toughness of Chit/Aga semi-IPN hydrogel and contributing to the excellent energy dissipation and stress bearing upon deformations. As shown in Figure 8, it was observed that after subjecting the hydrogels to external compressions, they were plastically deformed as expected. However, the hydrogels were able to withstand the compression without any obvious damage and demonstrated fully reversible recovery when the stress was removed, due to the reversible collapse of the inter-connected pores within the gel matrix. This IPN structure incorporated hydrogel possessed good mechanical integrity and good resilience for shape recovery, further validating the excellent properties of the hydrogels for tissue patch applications.

### 3.6. Drug Release Behavior

**Figure 9:**
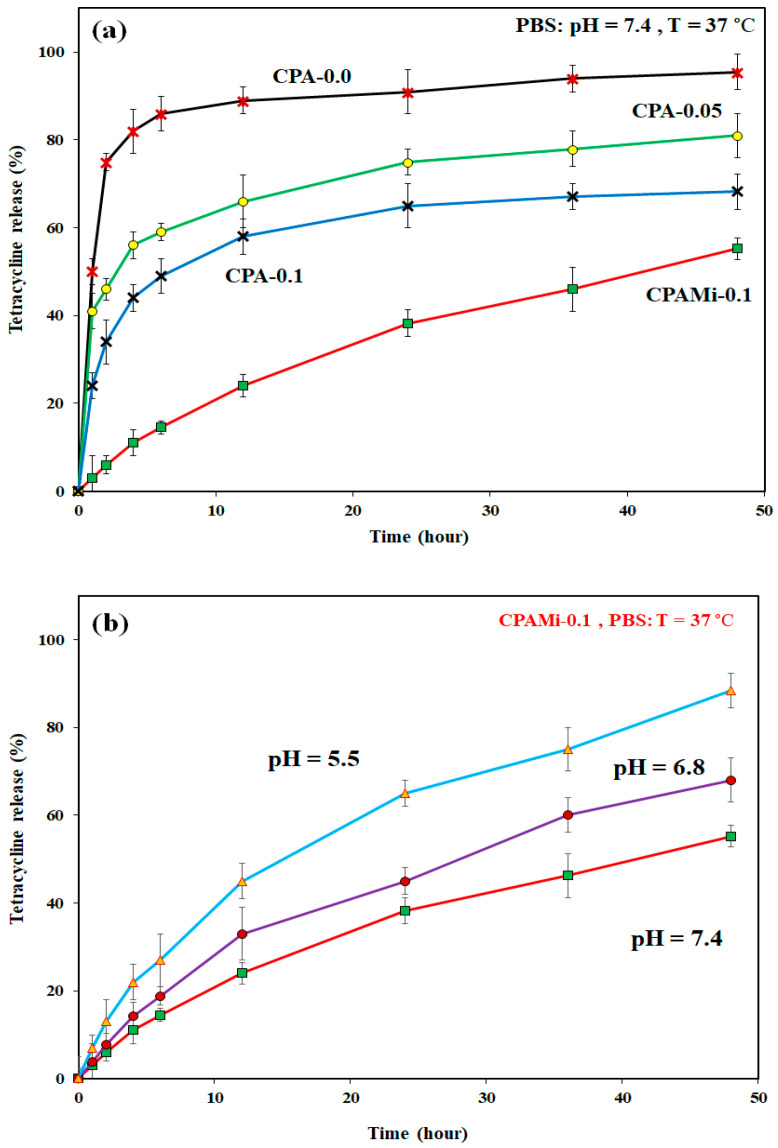
(**a**) Release profile of tetracycline from CPA-based hydrogels in PBS (pH: 7.4) at 37 °C. (**b**) Release profile of tetracycline from CPAMi-0.1 hydrogel in different pH solutions at 37 °C.

A wound dressing should have a controlled drug-release ability to avoid frequent replacement and lower the risk of overexposing the wound to bacteria. Therefore, this study is conducted to take advantage of Plr and Alg beads on controlled delivery of TC. For in vitro release experiments, TC release from CPA-0.0, CPA-0.05, CPA-0.1, and CPAMi-0.1 was analyzed in PBS with different pHs at 37 °C, and the results are displayed in Figure 9a,b.

#### 3.6.1. Drug Release Behavior of Different Formulations

In the CPA-0.0 formulation, a biphasic drug release profile was seen with the early sudden release of 86% (during the first 6 h) followed by a slower release phase. After 48 h, the cumulative release of TC reached a value of 95.5%, which was much higher than other formulations. One factor contributing to the drug’s initial rapid release was its adhesion to the gel’s surface, where it was easily released in PBS. Since the hydrogel lacks covalent cross-linking to encapsulate TC, diffusion could be the possible factor that affected the release profile of TC. Easy hydration of Chit and Aga, water diffusion into the gel, and rapid swelling might be important factors. After hydration, hydrophilic low molecular weight medicines can quickly diffuse to dissolution aqueous medium across the interconnected porous structure, initiating an initial burst release. As a result, the drug release rate is controlled by an increase in volume during the hydration process, which in turn can be controlled by the composition of the hydrogels [8,13]. Therefore, by employing Plr to enhance the rigidity of the hydrogel network, the release of drugs may be further controlled.

Compared with CAP-0.0, formulations containing Plr demonstrated superior sustained release effects and the release of TC decreased as the concentration of Plr increased. The cumulative release of TC from CPA-0.05 and CPA-0.1 gradually came to about 59% and 48% after 6 h, respectively. After 48 h, the cumulative release reached 81% and 68%. The arrangement of Pluronic (Plr) within the hydrogel matrix plays a crucial role in determining the porosity of the hydrogel microstructure, which directly influences the diffusion of drugs into the surrounding environment. As the concentration of Pluronic increases, the interaction between the polymer chains becomes more pronounced, resulting in a more compact and entangled network. This increased entanglement reduces the available pore size, leading to higher tortuosity in the aqueous channels and creating a more complex diffusion pathway for the drug. Consequently, the drug release rate is reduced, as the more intricate structure limits the contact between the drug and the release medium. These findings demonstrate that the arrangement of Pluronic within the hydrogel matrix acts as an additional barrier to drug diffusion, providing a more controlled and sustained release [2,14,44].

To further control the release, a sample containing Alg beads was also prepared. The lack of an initial burst effect in the release profile suggests the drug incorporation into the beads and the absence of poorly adsorbed drugs on the hydrogel surface. Drug molecules were trapped in cross-linked Alg beads, and the cross-linked polymer chains reduced the diffusion coefficient and prevented the release of the drug molecules [10,12]. It was clear that the formulation containing cross-linked beads had a superior potential for sustained release and had been successful in delaying the release up to 48 h (55%) compared to CPA-0.0 hydrogel (95.5%) (*p* < 0.05). CPAMi-0.1 hydrogel has the best ability of sustained release effect, which is required for a wound dressing, due to the dual obstacles of beads and hydrogels.

#### 3.6.2. Drug Release Behavior at Different pHs

Figure 9b shows the hydrogel release at different pH. As discussed earlier, a low pH promotes the protonation of primary amines. At pH 5.5 chitosan exists as polycation, leading to swelling of polymeric matrix. This will allow drug molecules to move more freely through normal diffusion. This can be seen from TC release at pH 5.5 where release reached approximately 74% in the first 48 h, which is significantly higher than the release at neutral pH (*p* < 0.05). Also, there was no significant difference in the release profiles evaluated at pH 5.5 and 6.8. A possible drug release mechanism for CPAMi hydrogels was proposed and depicted in Scheme 2.

### 3.7. Antibacterial Performance

One of many factors that affect skin wound healing is infection by bacteria including *S. aureus*, *P. aeruginosa*, *S. saprophyticus*, and others. Therefore, the antimicrobial activity of CPAMi-0.1 hydrogel was investigated. Results of antimicrobial activity of composite hydrogel and all relevant controls were presented in Table 1. The outcomes obtained revealed that the number of bacteria inhibited by hydrogel was variable, of which *S. saprophyticus*, *E. faecalis* and *C. albicans* (12.5 μg/mL) were significantly inhibited, and *P. aeruginosa* (100 μg/mL) was partially inhibited, and *S. aureus*, *E. coli*, *K. pneumoniae*, and *S. typhimurium* (200 μg/mL) were weakly inhibited by hydrogel. However, MBC was not detected against the bacteria used. As seen composite hydrogel showed greater inhibitory properties against Gram-positive bacteria than Gram-negative bacteria, possibly due to the cellular envelope structure in Gram-negative bacteria, because these bacteria have additional outer membrane and porin or porin-like proteins in their envelope structure. The presence of these structures in Gram-negative bacteria gives them the ability to selectively enter hydrophobic and hydrophilic molecules inside the bacteria, thus protecting the bacterial cell from antibacterial substances.

Other studies suggest that the presence of Chit may also contribute to antibacterial activity. Chit’s antibacterial activity is mostly derived from its cation groups and is dependent on its molecular. The poly-cationic Chit can interact with the electronegative surface of bacteria, enhance the permeability of the bacteria cell wall and, accordingly the leakage of intracellular constituents and the dissipation of ionic gradients within bacteria. Entrapment of TC in composite hydrogel has potentiated Chit antimicrobial activity against bacteria. As an external medical device, this dressing was effective against bacteria and met the criteria for antimicrobial characteristics.

### 3.8. In Vitro Evaluation of Hydrogel Cytotoxicity and Hemocompatibility

**Figure 10:**
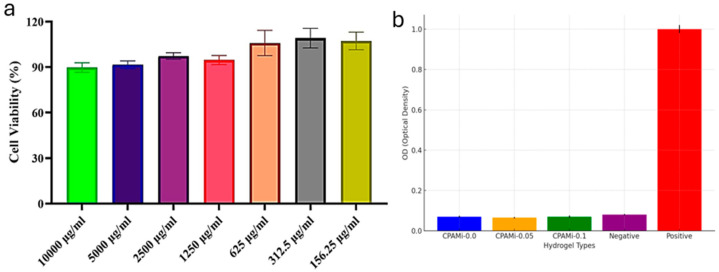
(**a**) Biocompatibility study of developed hydrogels (CPAMi-0.1) using MTT assay. (**b**) Hemocompatibility of hydrogels.

Despite all the advances in the development of polymeric-based hydrogels, there are still concerns regarding the potential adverse effects of hydrogels on healthy cells. As a matter of fact, an ideal hydrogel for the purpose of in vivo and in vitro usage should not impose any toxicities on cells. Consequently, the biocompatibility of the hydrogels was evaluated with the HFF2 cell line utilizing the MTT method. As shown in Figure 10a, according to the MTT results, composite hydrogel over a broad variety of concentrations had no deleterious effects on HFF2 cells even at a high concentration of (10 mg/mL). Indeed, the cell viability rate did not decrease considerably by increasing the hydrogel concentration. The survival rate was close to 100% or even beyond in concentrations lower than 2.5 mg/mL while hydrogels at a concentration of 10 mg/mL exhibited a 90% cell survival rate [45]. From the ANOVA test, there were no statistical differences (*p* < 0.05). The hemocompatibility of the developed hydrogels was assessed by measuring the optical density (OD) as an indicator of red blood cell lysis in contact with the hydrogel samples. As depicted in Figure 10b, all hydrogel formulations (CPAMi-0.0, CPAMi-0.05, and CPAMi-0.1) exhibited low OD values, similar to the negative control, indicating negligible hemolytic activity. In contrast, the positive control showed a significantly higher OD value, suggesting substantial red blood cell damage. The low OD values for all hydrogel types confirm that they do not induce hemolysis and are therefore hemocompatible.

## 4. Conclusions

The prepared CPA-based hydrogel that was loaded with Alg beads and TC is a safe, convenient and effective wound dressing. Chit, a widely known wound dressing material, was mixed with Aga and Plr, resulting in the development of a composite hydrogel. The interactions between these polymers brought improvement in mechanical performance in comparison to the pure polymer. By increasing the concentrations of Plr in hydrogels to an appropriate value, the porosity, swelling ratio and degradation rate of composite dressing were markedly improved. The TC-loaded hydrogel provided the desired sustained release ability over a period of almost 2 days, due to a double drug delivery approach of embedding Alg beads into the composite hydrogel. This hydrogel dressing demonstrated excellent growth inhibition effects against *S. saprophyticus*, *E. faecalis*, and *C. albicans* as well as excellent biocompatibility on HFF2 cells. The findings suggest that this developed composite dressing fabricated in this study may have great potential for use as a dressing material to enhance wound healing.

## Figures and Tables

**Table 1:** MIC and MBC of CPAMi-0.1 hydrogel against range of pathogenic bacteria.

Organism	ATCC	MIC (μg/mL)	Negative Control	Positive Control
*S. aureus*	29213	200	Absence of growth	Growth of bacterium
*S. saprophyticus*	15305	12.5	Absence of growth	Growth of bacterium
*E. faecalis*	29212	12.5	Absence of growth	Growth of bacterium
*E. coli*	25922	200	Absence of growth	Growth of bacterium
*K. pneumoniae*	7881	200	Absence of growth	Growth of bacterium
*S. typhimurium*	14028	200	Absence of growth	Growth of bacterium
*P. aeruginosa*	27853	100	Absence of growth	Growth of bacterium
*C. albicans*	10231	12.5	Absence of growth	Growth of yeast

## Data Availability

The original contributions presented in the study are included in the article, further inquiries can be directed to the corresponding author.
